# Beyond Lipid Signaling: Pleiotropic Effects of Diacylglycerol Kinases in Cellular Signaling

**DOI:** 10.3390/ijms21186861

**Published:** 2020-09-18

**Authors:** Jae Ang Sim, Jaehong Kim, Dongki Yang

**Affiliations:** 1 Department of Orthopedic Surgery, Gil Medical Center, College of Medicine, Gachon University, Incheon 21565, Korea; sim_ja@gilhospital.com; 2 Department of Biochemistry, College of Medicine, Gachon University, Incheon 21999, Korea; geretics@gachon.ac.kr; 3 Department of Physiology, College of Medicine, Gachon University, Incheon 21999, Korea

**Keywords:** diacylglycerol kinase, diacylglycerol, phosphatidic acid, tissue microenvironment, tumor microenvironment, lipid signaling

## Abstract

The diacylglycerol kinase family, which can attenuate diacylglycerol signaling and activate phosphatidic acid signaling, regulates various signaling transductions in the mammalian cells. Studies on the regulation of diacylglycerol and phosphatidic acid levels by various enzymes, the identification and characterization of various diacylglycerol and phosphatidic acid-regulated proteins, and the overlap of different diacylglycerol and phosphatidic acid metabolic and signaling processes have revealed the complex and non-redundant roles of diacylglycerol kinases in regulating multiple biochemical and biological networks. In this review article, we summarized recent progress in the complex and non-redundant roles of diacylglycerol kinases, which is expected to aid in restoring dysregulated biochemical and biological networks in various pathological conditions at the bed side.

## 1. Introduction

**Figure 1:**
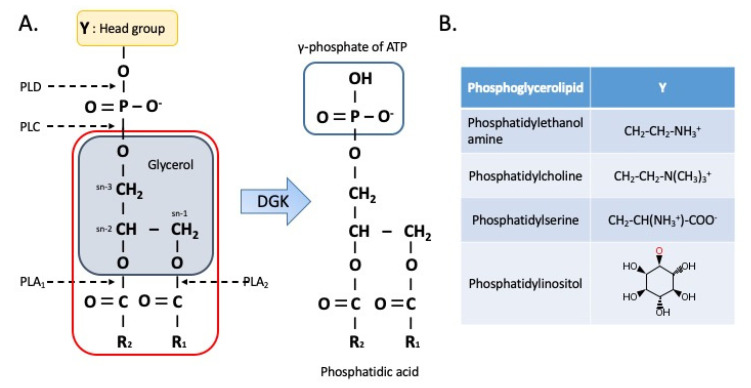
Structure of phosphoglycerolipid including diacylglycerol (DAG) and phosphatidic acid (PA). (**A**) The sites for phospholipase-mediated hydrolysis of phosphoglycerolipid are marked in letters. Structure of DAG is presented in a rounded red rectangle. (**B**) The head groups (Y) of selected phosphoglycerolipid classes are presented. Y is ethanolamine, choline, serine and inositol from top to bottom. O in red indicates hydroxyl group of phosphoglycerolipid where the inositol residue is bound. ATP, adenosine triphosphate; DGK, diacylglycerol kinase; PLA, phospholipase A; PLC, phospholipase C; PLD, phospholipase D. R_1_ and R_2_ are fatty acid residues.

The addition or removal of phosphate group in lipids or proteins is critical for cellular signal transduction. Diacylglycerol kinases (DGKs, EC 2.7.1.107), which are involved in the phosphoinositide signaling pathway, mediate the cellular responses to abiotic and biotic stresses. In the DGK-catalyzed reaction, γ-phosphate of adenosine triphosphate (ATP) is transferred to the hydroxyl group of sn-1,2 diacylglycerol (DAG), which promotes the synthesis of phosphatidic acid (PA) from DAG (Figure 1). DGKα was first purified from the pig brain in 1983 [1]. The human DGKα cDNA was cloned from the leukocytes in 1990 [2]. In 1994, human DGKα was genetically mapped to chromosome 12 [3]. Currently, 10 mammalian DGK isozymes have been identified (α [2,4], β [5], γ [6,7], δ [8], η [9], κ [10], ε [11], ζ [12], ι [13], and θ [14]). The activity of DGK is detected in various tissues and organisms (from *Escherichia coli* to mammals) [15,16,17]. The number of DGK isoforms varies from yeast (single enzyme) to mammals (10 isozymes, some with splicing variants) and the specialized activity of DGK increases with the complexity of organisms. The phenotypes of DGK knockout (KO) mouse and the disease-associated changes in the expression and activity of DGKs indicate that DGK isoforms exhibit specialized non-redundant functions [16].

**Figure 2:**
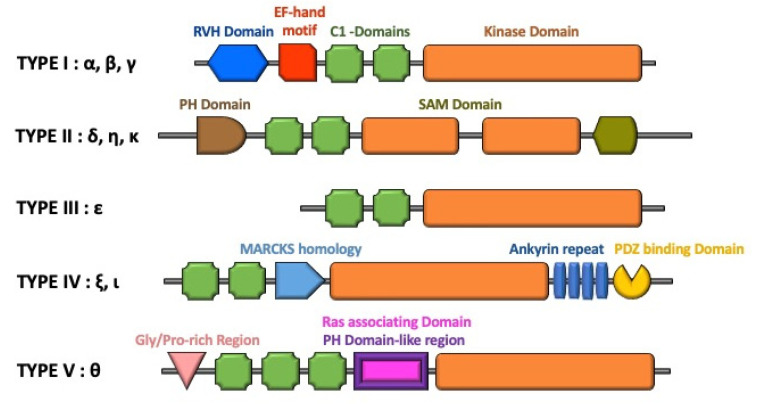
The structures of DGKs. DGK isoforms are classified into five types. Gly/Pro, glycine/proline; PH, pleckstrin homology; RVH, recoverin homology domain; MARCKS, myristoylated alanine rich protein kinase C substrate phosphorylation site; SAM, sterile alpha motif.

The structures of DGK isoforms are presented in Figure 2. The mammalian DGKs, which have at least two cysteine-rich C1 domains (C1a and C1b domain) for interacting with DAG and one kinase domain with catalytic and accessory subdomains, represent a large enzyme family. The ten isoforms of mammalian DGKs are grouped into five types based on the homology of their structural features [18,19]. Type I DGKs (α, β, and γ) sequentially contain two calcium-binding EF-hand motifs (which enable the enzymes to respond to Ca^2+^ [20]), two C1 domains, and a catalytic domain. In the T cells, Ca^2+^ modulates the enzyme activity and also appears to localize DGKα activity [21]. Type II DGKs (δ, η, and κ) have an N-terminal pleckstrin homology (PH) domain that interacts with phosphatidylinositol (PI), two C1 domains, two catalytic domains, and finally, a C-terminal sterile α-motif (SAM) domain. Type III DGK (ε), which is the shortest DGK isoform, contains two C1 domains, followed by a catalytic domain. Type IV DGKs (ζ and ι) contain two C1 domains, followed by a myristoylated alanine-rich protein kinase C substrate phosphorylation site-like region (MARCKS homology domain), a catalytic domain, four ankyrin repeats, and a C-terminal PDZ-binding site. Type V DGK (θ) contains a proline- and glycine-rich domain, three C1 domains, a central PH domain, and a catalytic domain. A recent phylogenetic analysis of the conserved regions in the DGK catalytic domain of the main vertebrate classes and eukaryotic phyla demonstrated the evolutionary relationships between DGKs [22].

The elucidation of the physiological roles of DGKs has been challenging. The number of DGK isoforms varies in different mammalian tissues (at least one member of the DGK is expressed in all mammalian tissues and most DGK isoforms are abundantly expressed in the brain and hematopoietic cells) [23]. The analysis of expressed sequence tag data from the National Center for Biotechnology Information database containing the tissue expression pattern of DGKs revealed that the spectrum of DGK isoform expression is relatively narrow in several tissues [24].

The catalytic domains of the DGK isoforms effectively phosphorylate DAG through a regulated process. The differences in the activity of DGK isoforms are attributed to the structural variations in other domains, which determine the interaction with proteins that regulate the activity and subcellular localization of DGK isoforms. DGKs have kinase-dependent and kinase-independent functions [25].

At present, there is an important agenda to fulfill. The importance of different DGK isoforms (some of which share structural similarity) is unknown. These isoforms appear to exhibit non-redundant functions [26]. Thus, the evolutionary importance of DGK family enzymes with a low functional redundancy between the isoforms is not clear. It is important to identify the specific functions of different DGK isoforms localized in different subcellular compartments, such as the plasma membrane, endoplasmic reticulum (ER) and Golgi complex, cytoskeleton, endosomes, and nucleus. Additionally, the spatiotemporal regulation of DGK isoforms in the subcellular compartment must be examined. Furthermore, the therapeutic effects of DGK inhibitors on the tissue microenvironment, which comprises different types of epithelial, stromal, and immune cells, must be evaluated. Finally, DGK isoform-specific inhibitors must be identified.

## 2. Regulation of DAG and PA Levels

The DAG-dependent and PA-dependent signaling can be distinctly represented. However, both these signaling pathways are interconnected and they are essential for maintaining cellular homeostasis. Hence, this review will discuss various mechanisms that regulate the DAG and PA levels in the eukaryotic cells, especially in mammals to maintain the homeostasis of cells and organs.

DGKs are one of the pivotal enzymes in lipid signal transduction. Since DAG is primarily metabolized by DGKs to produce PA, DGKs regulate multiple signaling pathways through the attenuation of DAG signaling and initiation of PA signaling pathways. Both DAG and PA can serve as important secondary messengers [27]. In contrast to PA, DAG moves freely across the trans-bilayer membranes in a process called “flip flop” without protein assistance [28].

**Figure 3:**
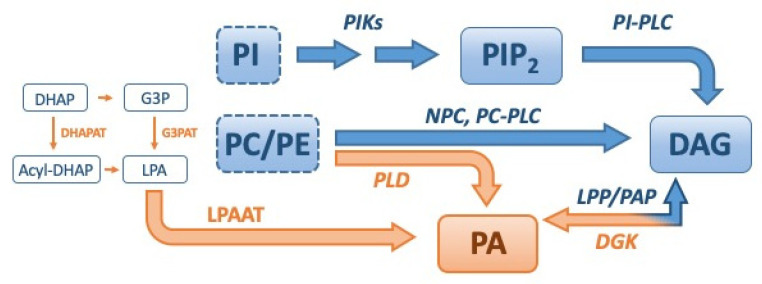
Representative pathways involved in the metabolism of diacylglycerol (DAG) and phosphatidic acid (PA). The biosynthetic pathways of DAG and PA are shown in blue and orange, respectively. The key enzymes of the DAG and PA biosynthetic pathway are shown in blue and orange italics, respectively. The enzyme inhibitors are represented in green font. The pathways involved in the degradation of DAG and PA are shown in black font. AT, acyltransferase; DHAP, dihydroxyacetone-3-phosphate; G3P, glycerol-3-phosphate; LPA, lysophosphatidic acid; LPAAT, LPA acyl transferase; LPP, lipid phosphate phosphatase; NPC, non-specific phospholipase C; PAP, phosphatidic acid phosphatase; PC, phosphatidylcholine; PE, phosphatidylethanolamine; PI, phosphatidylinositol; PIK, phosphatidylinositol kinase; PLC, phospholipase C; PLD, phospholipase D.

Representative pathways for DAG regulation are illustrated in Figure 3. The chemical structures of DAG species are highly variable and DAGs represent a class of lipid molecules exhibiting various isomeric properties. Glycerol-3-phosphate (G3P) is the basic building block of every phospholipid (PL) with a phosphate residue at the sn-3 position. Owing to the sn-3 position of the phosphate residue in PLs, DAGs generated from the hydrolysis of the PL headgroup or the dephosphorylation of PA by phosphatic acid phosphatases (PAPases, LIPINs) are per definition sn-1,2 isomers (Figure 1 and Figure 3) [29,30]. The DAG pool in the plasma membrane and ER/Golgi network is reported to comprise sn-1,2 DAGs [29,30]. The selective substrate for DGKs is sn-1,2 DAGs [29], while that for DGKε is DAG with an arachidonoyl group (C20:4), which is the main product of phosphatidylinositol phospholipase C-mediated phosphoinositide hydrolysis at the sn-2 position [31]. In addition to the plasma membrane, DGK isoforms are involved in the DAG/PA metabolism in the nucleus, ER/Golgi complex, and cytoskeleton [24,29,32].

Protein kinase C (PKC) is activated by sn-1,2 DAG [18,33,34]. Additionally, PKC interacts with various protein targets, including the protein kinase D family members [23,35], Ras guanyl nucleotide-release proteins (RASGRP: exchange factors for Ras/Rap1) [34], chimaerins (a family of Rac GTPase-activating proteins) [36], Munc13 family (scaffolding proteins involved in exocytosis) [37], and some transient receptor potential canonical channels (Ca^2+^-permeable nonselective cation channels) [38]. Thus, the DGK-mediated hydrolysis of DAG regulates various cellular processes. DAG can also regulate the membrane translocation and activity of its interacting proteins by binding to their C1 domains [39,40].

PA localizes to various subcellular compartments, such as the plasma membrane, ER, Golgi complex, mitochondria, and nucleus [41], and interacts with the putative PA-binding regions, whose consensus sequence has not been characterized, of more than 50 different proteins [42,43]. Additionally, PA regulates various signaling proteins, including mammalian/mechanistic target of rapamycin (mTOR) [44], phosphatidylinositol 4-phosphate 5-kinase [45], PKC-ζ [46], protein phosphatase 1 [47], Src homology region 2 domain-containing phosphatase 1, ArfGAP, Arf, Raf1 [48], DEP (disheveled (Dsh), Egl-10, and pleckstrin) domain of Epac1 [49], Dsh proteins [50], phospholipase C-γ1 [51], IQGAP1, and PIP5-kinase (PIPKIα) [52]. Furthermore, PA modulates cell survival and proliferation, cytoskeletal organization, and membrane and vesicle trafficking [53].

In addition to DGKs, various enzymes catalyze the biosynthesis of PA [54]. For example, de novo synthesis through the sequential acylation of G3P and lysophosphatidic acid, which is the main metabolic route catalyzed by lysophosphatidic acid acyltransferase (LPAAT) and phospholipase D (PLD)-catalyzed hydrolysis of PLs, such as phosphatidylcholine (PC) or phosphatidylethanolamine (PE) are shown in Figure 3. The detailed biochemical and biological properties of DAG and PA species are excellently presented elsewhere by Eichmann [29] and Pokotylo [43], respectively.

## 3. Regulation of DGK Isoforms

### 3.1. Phospholipids

PLs can upregulate the activity of DGK isoforms [55]. The activity of DGKα is upregulated by PC, PIP3, and PI(3,4)P2 [1]. DGKβ is activated by PI(4,5)P2 and phosphatidylserine (PS) [56]. The activities of type I DGKs are dependent on Ca^2+^ and PS [55,57]. Treatment with DGKα inhibitors is reported to reduce the intracellular Ca^2+^ levels, which indicated that Ca^2+^ and DGKα are a part of a positive feedback loop [58]. PA activates DGK isoforms with high efficiency, which indicates the presence of a positive feedback mechanism [59,60,61]. PS treatment does not activate DGKδ, while constitutive RhoA overexpression inhibits the activity of DGKθ [60,62]. PIP2, a precursor of DAG, inhibits the activity of DGKθ [63].

### 3.2. Subcellular Localization

The plasma membrane translocation has been suggested to determine the spatiotemporal activity of DGK isoforms. Treatment with phorbol ester induces the plasma membrane translocation of DGKδ [64], which is regulated by the classical PKC (cPKC)-catalyzed phosphorylation of Ser-22 and -26 in the PH domain [65]. Expression pattern and subcellular localization of DGK isoforms are presented in Table 1.

The type II DGK (δ and η) isozymes share a protein-protein interacting region, called SAM domain. The dynamic polymerization of the SAM domain inhibits the plasma membrane translocation and the activity of DGKδ [66]. Agonist-induced phosphorylation regulates the translocation and activation of some DGK isoforms. Ca^2+^ treatment [20], purinergic receptor stimulation [67,68], and T cell receptor (TCR) stimulation [69] translocate DGKα to the plasma membrane. The plasma membrane translocation of DGKα is also dependent on the phosphorylation of Tyr-334 of human DGKα (Tyr-335 in mouse DGKα) catalyzed by Src family tyrosine kinases (Src and Lck) [70], while the nuclear export of DGKα is dependent on the phosphorylation of Tyr-218 catalyzed by Src-activated c-Abl tyrosine kinase [71]. The vitamin E-induced phosphorylation of DGKα, which is catalyzed by Src family tyrosine kinases, promotes the plasma membrane translocation and the activity of DGKα [72,73]. DGKα is transported to the nucleus from the cytoplasm under serum starvation conditions. Although the nuclear function of DGKα is not clear, the nuclear DGKα activity was reported to promote the proliferation of K562 cells by promoting the G1/S transition [74]. Additionally, DGKα stabilizes Src activation, which indicated that DGKα and Src are a part of a positive feedback loop [75]. Previous studies have also reported the translocation of DGKα [76], DGKγ [77], DGKδ [60], DGKζ [78], DGKθ [60], and DGKι [79] from the cytosol to the nucleus.

### 3.3. Transcriptional Control

The expression of DGKα and DGKζ is regulated at the transcriptional level [94]. The promoter of the DGKα gene contains a binding site for early growth response 2 (EGR2), which is a transcription factor (TF) that is upregulated upon TCR stimulation [95]. The upregulation of DGKα expression is important for T cell anergy [96]. Forkhead box O1 and 3 (FoxO1 and 3) TFs bind to the promoter of DGKα in the quiescent T cells. TCR stimulation decreases binding of FoxO TFs to the DGKα promoter, which results in decreased transcription of DGKα. DGKζ expression is also upregulated in the anergic cells through EGR2 [97]. DGKζ, which lacks the calcium-binding EF motif, is nonresponsive to Ca^2+^ signals and is activated through PKC-mediated phosphorylation. The nuclear export of DGKζ is dependent on the PKCα-mediated phosphorylation of its MARCKS homology domain [78,98].

### 3.4. Post-Translational Control

Norepinephrine stimulates the activity of DGKθ through a PI3K-dependent mechanism [99]. In the human adrenocortical cells, steroidogenic factor 1 and sterol regulatory element-binding protein 1 mediate the cyclic adenine monophosphate (cAMP)-induced expression of DGKθ [100]. The phosphorylation of Ser-776 and -779 in the accessory subdomain of DGKγ is catalyzed by PKCγ after purinergic stimulation, which enhances the enzyme activity [68]. PKCγ directly interacts with DGKγ through its accessory subdomain, which is dependent on Ca^2+^, PS, and diolein. DGK negatively regulates PKC activation by downregulating the level of DAG. Polybasic proteins such as histone H1 and Tau stimulate DAG binding and catalytic activity of DGKθ [61].

### 3.5. Inhibitors

R59022, R59949, and ritanserin are allosteric inhibitors of DGKα [101]. They are also serotonin receptor antagonists. R59949 strongly inhibits type I DGKs (α and γ) and moderately inhibits type II DGKs (θ and κ) [102]. The half-maximal inhibitory concentration (IC50) values of R59022 and R59949 against DGKα are 25 and 18 µM, respectively [103]. R59022 and R59949 share a highly similar structure and the structure of R59022 differs by a single fluorine from ritanserin. The IC50 value of ritanserin against DGKα is approximately 20 µM. Recently, CU-3, a competitive ATP inhibitor, was identified as a novel DGKα-selective inhibitor (IC50 = 0.6 µM). The administration of CU-3 enhanced cancer cell apoptosis and immune responses in vitro [103,104]. However, the structure and reactivity of CU-3 are not suitable for in vivo applications. AMB639752 was recently identified as a novel DGKα-selective inhibitor, devoid of serotoninergic activity, from an in-silico approach [105].

Hence, there is an urgent need to identify novel, specific, and potent small-molecule inhibitors of DGK isoforms. There are ongoing efforts to identify these inhibitors. However, the identification of DGK inhibitors is challenging because the catalytic domain of mammalian DGK has not yet been crystallized and the active sites of DGK are poorly characterized. The lack of crystal structures has seriously limited the mechanistic understanding of the interaction between substrate and inhibitor. Recently, a proteomic study identified the novel ligand-binding regions that mediate the binding of ATP and inhibitor to the poorly characterized active site of DGK isozymes [106]. Additionally, another study reported the crystal structure of EF-hand domains of DGKα in its Ca^2+^-bound form, which can regulate intra-molecular interactions [107].

## 4. Signaling Pathways Regulated by DGK Isoforms

Because DGK isoforms regulate multiple signaling pathways through the attenuation of DAG signaling and initiation of PA signaling pathways, the biological functions of DGKs can result from both DAG hydrolysis and enhanced PA biosynthesis. However, further studies are needed to delineate the processes responsible for the specific biological functions of DGKs.

**Figure 4:**
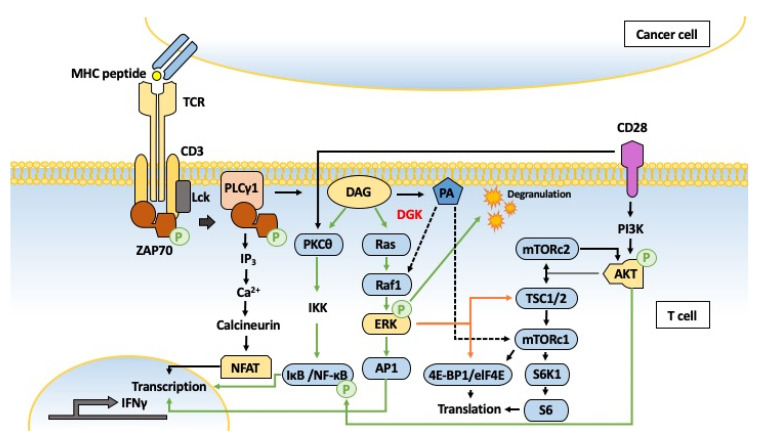
Representative signaling pathways regulated by diacylglycerol kinases (DGKs) in the activated T cells. Our understanding about diacylglycerol (DAG)-mediated regulation of Ras-MEK-ERK and PKC-NF-κB is majorly based on T cell biology. Immunological synapse formation between T cell and cancer cell is shown as a representative model here. Signals originating from T cell receptor (TCR) engagement of peptide/MHC complex in the presence of co-stimulatory signal lead to the recruitment of adaptor molecules and degranulation, which promote the lysis of the target cells and secretion of IFNγ (T cell effector function). DGKs downregulate the levels of DAG, which activates the TCR distal signaling through Ras-ERK and NF-κB (green arrow). The biosynthesis of phosphatidic acid (PA) in the T cells is mediated by DGKs and phospholipase D (PLD) (not shown). PA is shown to activate Raf1 and mTORC1 in the immune and non-immune cells (dotted black arrow).

The summary of representative DGK-regulated signaling pathways is shown in Figure 2. Activated PLCγ1 cleaves PIP2 in the plasma membrane to generate two secondary messengers, DAG and IP3. DAG activates PKC, AP-1 and partly NF-κB and IP3 is involved in the activation of intracellular Ca^2+^ flux. The upregulated Ca^2+^ signaling activates the TF and NFAT. In the T cells, the antigen-stimulated DAG production is dependent on the strength of antigenic stimulation and determines the duration and intensity of the Ras/MEK/ERK and PKC-dependent signaling pathways (Figure 4).

### 4.1. Ras/MEK/ERK Pathway

DGKα and DGKζ negatively regulate T cell response and induce T cell anergy [108,109,110]. IL-2 suppresses DGKα to restore the responsiveness in the anergic T cells [111]. DGKα and DGKζ exhibit both redundant and specialized functions in the T cells, which limit the intensity of DAG-regulated signals downstream of the antigenic stimulation [112]. The binding of Ca^2+^ and PIP3 to EF-hands and C1 domain, respectively, and Src family kinase (Lck)-catalyzed tyrosine phosphorylation regulate the localization and activity of DGKα to the peripheral area of the immunological synapse. An immunological synapse is a specialized interface between a T cell and an antigen-presenting cell and consists of molecules involved in T cell activation. After the T-cell activation and CD-28 co-stimulation, the transcriptional suppression of DGKα is higher than that of DGKζ, which may be due to the PI3K-AKT signaling-mediated inhibition of FoxO-mediated transcription of DGKα [113]. The RASGRP/ERK pathway, which is activated by DAG, is crucial for the polarization of microtubule-organizing center in the cytotoxic T cells, delivery of lytic granules to the immunological synapse, and the subsequent lytic attack on the target cells [114]. Recent studies have focused on the functions of DGKα and DGKζ, which are involved in TCR signaling. The function of DGKδ in the T cells has not been elucidated [94].

DGK isoforms also regulate the Ras/MEK/ERK pathway in non-immune cells. In contrast to the T cells in which DGKα inhibition activates the Ras pathway, the inhibition of DGKα suppresses Ras in the hepatocellular carcinoma cells. This suggested the differential effects of DGKα inhibition on different cells and tissues [109,115]. DGKη, a critical regulatory component of the Ras/B-Raf/C-Raf/MEK/ERK signaling cascade, mediates B-Raf/C-Raf-dependent cell proliferation [116]. DGKδ positively regulates epidermal growth factor receptor (EGFR) signaling [117]. The DGKδ-deficient cells exhibit enhanced ubiquitination of EGFR owing to the decreased expression of ubiquitin-specific protease 8 (USP8), a deubiquitinase [118]. DGKι inhibits the RASGRP3-dependent Rap1 signaling pathway [119]. DGKγ suppresses Rac1 and regulates the formation of lamellipodium [120].

In the tumor microenvironment (TME), the tumor-suppressive function of resident immune cells, such as macrophages and T cells, are switched to pro-tumoral functions [121]. The anergic T cells and tumor-infiltrating T cells are reported to exhibit similar properties, such as enhanced DGKα levels, attenuated ERK responses (both low basal phosphorylation of ERK and low stimulation-induced phosphorylation of ERK and c-JNK), and downregulated expression of AKT and AKT client proteins (two regulatory factors of NF-κB: IκB and GSK3) [122]. The high level of DGKα and inhibited MAPK pathways can lead to the dysfunction of human tumor-infiltrating CD8+ T cells (pro-tumoral), which is reversible upon treatment with low-dose of IL-2 to suppress DGKα [123]. DGKζ is expressed in various hematological cells. The deficiency of DGKζ is reported to be immunostimulatory in the T cells, natural killer (NK) cells, and B cell but immunosuppressive or modulatory in the Treg cells, mast cells, macrophage, and dendritic cells [124].

### 4.2. NF-κB Signaling

DGKζ downregulates the p53-mediated cytotoxic responses [125] and cytokine-induced pro-inflammatory NF-κB signaling [126] in the HeLa cells. Additionally, DGK ζ downregulation shifts the limiting pool of CBP/p300 coactivators toward the NF-κB p65 subunit from p53, which indicated the presence of reciprocal antagonistic phenotypes between p53 and NF-κB [127].

### 4.3. Insulin Signaling

PI3Ks bound to phosphorylated Ser of activated insulin receptor substrate (IRS) induce the plasma membrane translocation of glucose transporter type 4 (GLUT4), which facilitates glucose uptake [128]. cPKC (α, β and γ) and novel PKCs (nPKCs; δ, ε, η, and θ) are activated upon binding with sn-1,2 DAG. Consequently, PKC-phosphorylated IRS does not serve as a scaffold for PI3K and decreases glucose uptake. Consistent with these mechanistic insights, the mouse models and human mutations associated with increased DAG levels exhibited hyperglycemia and insulin resistance [129,130,131,132,133,134]. The activation of PKCs only by sn-1,2 DAG indicates the importance of identifying DAG isomerism under physiological and pathological conditions to elucidate the correlation between DAG levels and DAG signaling mediated by DAG, DGK, and DAG-targeted proteins, such as PKC. DGKδ-mediated DAG degradation promotes the downregulation of PKC activity, which activates the insulin receptor signaling pathways [135].

### 4.4. Gα_q_ Regulation of PLC

In the non-immune cells, allosteric regulation of PLC-β1, which is activated by agonist-induced G protein-coupled receptor (GPCR), is required for its synergistic lipase-GAP activity. PA synthesized by RhoA-activated phospholipase D1 (PLD1) transduces signals through the unique PA-binding domain of PLC-β1 to potentiate Gα_q_-stimulated lipase activity of PLC. DAG-mediated PKC activity may limit the PA-mediated allosteric upregulation of PLC activity, which indicates that the complex regulation of PLC activity is dependent on the balance between DAG and PA [136,137].

### 4.5. Hypoxic Responses and Angiogenesis

Hypoxia promotes the accumulation of PA through the activation of DGKs [138]. The accumulated PA regulates the hypoxia-inducible factor (HIF)-1α level [138]. Consistent with this finding, R59949 treatment strongly impaired hypoxia-induced accumulation of HIF-1α [139]. The activation of DGKα activates both proliferative and migratory responses in the endothelial cells during vascular endothelial growth factor (VEGF)-induced angiogenesis [140]. R59949 treatment inhibits inducible nitric oxide production in the vascular smooth cells through the inhibition of L-arginine uptake [141]. Additionally, R59949 suppresses retinal neovascularization and protects the retinal astrocytes in an oxygen-induced retinopathy model [142]. This further suggested the role of DGKs, especially DGKα, in regulating angiogenesis. Under hyperglycemic conditions, de novo synthesis of DAG activates PKC in the glomerulus, which activates the vicious cycle of PKC-ROS loop [143].

### 4.6. Endosomal Trafficking

Recently, DGKs were reported to regulate endosomal trafficking [144]. DGK isoforms regulate various stages of the endocytic trafficking, through the metabolic conversion of DAG to PA, including endocytosis, recycling endosomes, recycling of integrin and MHC complex, transporting from/to the Golgi apparatus, and the formation and secretion of multivesicular bodies [145,146,147,148]. DAG regulates membrane trafficking through the following mechanisms: activation of PKC, PKD, and downstream signaling cascades [149,150,151,152]; regulation of PI cycle to maintain the levels of PIP2, IP3, and Ca^2+^ influx; promoting membrane fusion and fission through the conical-shaped DAG provision of negative membrane curvature [153,154]. The spatial concentration of PA is also an important regulator of trafficking [144]. Recent studies have suggested a novel mechanism for the limited PKCα activation in the T cells, which involves the interaction between DGKζ and Sorting Nexin 27 [155]. This suggested that DGKζ deficit may also contribute to the pathogenesis of Alzheimer’s disease through the dysregulation of PKC activity. DGKζ-mediated activation of Rac1, a Rho GTPase family protein, plays a pivotal role in macropinocytosis and phagocytosis [156].

### 4.7. mTOR Signaling

In addition to DGKs, various enzymes catalyze the biosynthesis of PA [54]. For example, PA is produced from PLD-catalyzed hydrolysis of PLs, such as PC or PE (Figure 3). PLD-generated PA (PLD-PA) is one positive input for mTOR signaling [157]. PA-dependent activation of mTORC1 is mediated through the Raf-ERK pathway [158] or direct binding of PA to mTORC1, followed by displacement of inhibitory FKBP38 from mTORC1, and allosteric stimulation of the catalytic activity of mTORC1 [159]. In the T cells, TCR engagement and DAG activate the mTOR signaling through mTORC1 and 2 from the Ras-MEK1/2-ERK1/2 pathway. DGKα and ζ synergistically inhibit TCR-induced mTOR activation by inhibiting the Ras-MEK1/2-ERK1/2 pathway [160]. Thus, DGKs can inhibit mTOR signaling and both DAG and PA function as agonists of mTOR signaling in the T cells.

### 4.8. Different Effects of PAs Produced by DGK and PLD

At the sn-1 and -2 positions, DAG and PA contain different acyl chains. Thus, some studies have suggested that mammalian cells have more than 50 structurally distinct DAG/PA species [27]. However, the effect of different DAG/PA species on various cellular signaling events is unclear. The major PA species produced by PLD activity is 36:1 PA [161], while those produced by DGKs are 38:3, 38:4, and 38:5 PA [147]. Various PA species are reported to exhibit differential degrees of interaction and/or activation of the target proteins.

For example, Rab-coupling protein (RCP) must bind to PA to be tethered at the tips of invadopodia. The inhibition of DGKα inhibited the recruitment of RCP to allow α5β1 integrin recycling and promote the invasiveness of tumor cells, while the inhibition of PLD, which also generates PA, did not affect its recruitment [147]. Thus, DGKα is more important than PLD for RCP functions as a source of PA. DGK-generated PA (DGK-PA) is also involved in anti-neutrophil cytoplasmic antibody (ANCA)-driven granule exocytosis and adhesion of leukocytes [162,163].

PLD-PA is reported to be involved in cell invasion and tumor metastasis. The spatial production of PA by PLD is important for regulating microtubule-based and kinesin-based trafficking of endosomes containing prometastatic MT1-MMP for the secretion at invadopodia [52]. The PLD-catalyzed PA production is mediated through the hydrolytic cleavage of PC or PE. PLD-PA directly binds LATS and NF2, inhibits LATS-MOB1 complex formation, and promotes NF2-mediated membrane translocation and activation of LATS [164]. This PLD-PA driven inhibition of Hippo signaling activates the oncogenic Yes-associated protein and transcriptional coactivator with PDZ-binding motif (YAP/TAZ) signaling (PLD-PA-YAP signaling axis). Although a DAG analog, 12-O-tetradecanoyl-phorbol-13-acetate upregulates YAP/TAZ signaling through PKC activation [165], the role of DGK-PA in regulating YAP/TAZ signaling is not reported. These findings indicate that the increased or decreased pool of PA produced from the upregulation of DGKs or LIPINs may not regulate YAP/TAZ signaling.

## 5. Pathological Manifestations

Studies on DGK KO mice demonstrated the importance of DGKs in the immune system [96], and their pathophysiological roles in the brain and heart [166], insulin resistance in diabetes [167], and Friedrich’s ataxia [168]. *Toxoplasma gondii*-secreted DGK orthologs were demonstrated to function as key upstream activators of the programmed egress of the parasite from the host cells [169]. Recent studies have demonstrated the differential roles of several DGK isoforms in human diseases. DGK deficiency is directly or indirectly involved in human diseases, such as cancer, atypical hemolytic uremic syndrome, hypospadias (DGKκ) [170], diabetes, and mood and cognitive disorders, including Parkinson’s disease and fragile X syndrome, a primary cause of autism and inherited cognitive disorder [168]. Currently, the closest association between the loss-of-function mutation and human disease appears to be observed from DGKε [16]. Recessive mutations of DGKε are associated with atypical hemolytic uremic syndrome, a disease characterized by renal vascular endothelial injury due to diminished poly-unsaturated fatty acids, which are required for prostanoid synthesis [171,172]. Genome-wide association studies (GWASs) have also revealed the correlation between diseases and other DGK isoforms [19].

### 5.1. Cancer

DGKs are reported to be involved in cancer progression. Several studies have reported that DGKα is involved in cancer progression. However, the function of DGKα in cancer is controversial as it might also function as a tumor suppressor. DGKα exhibits pro-tumoral, anti-immunogenic, and fibroblast-activating properties, which indicated that selective inhibitors of DGKα can simultaneously exert antitumoral and pro-immunogenic effects in the TME [19,96,173,174,175]. The pro-tumoral hypoxic and immunosuppressive TME upregulates DGKα in the cancer and infiltrating T cells. Additionally, DGKα stabilizes oncogenic Src activation. The upregulated DGKα expression is required for the growth and survival of highly metastatic cancer cells but not for those of non-transformed primary cells [75]. However, recent studies have also demonstrated the anti-tumoral effect of DGKα in different cancer types [16,176,177,178]. Thus, DGKα appears to be both pro- and anti-tumoral depending on cancer cell-context.

Additionally, some cancers are dependent on DGKα-regulated pathways, which promote cancer cell survival [179]. DGKα, a positive regulator of NF-κB, suppresses tumor necrosis factor-α-induced apoptosis in the human melanoma cells through the activation of NF-κB [180]. The pro-tumoral function of DGKα is not reported for other DGK isoforms.

Decreased DNA methylation at the enhancer region of DGKα promotes the recruitment of the profibrotic TF, EGR1, and radiation-induced transcription of DGKα, which results in the profibrotic pathway activation in the dermal fibroblasts derived from patients who later developed fibrosis [175]. The role of DGKα in the generation of cancer-associated fibroblasts in the TME has not been elucidated. The analysis of epigenetic silencing revealed that the methylation of the DGKγ promoter is positively correlated with KRAS and BRAF mutations and that the methylation is frequently observed in colorectal adenomas, which suggested the significance of methylation in early colorectal tumorigenesis [181]. Consistent with this finding, the downregulated expression of DGKα synergizes with oncogenic mutations of p53 and Ras genes to promote transformation [16,176,177,178].

It has been shown that DGKι and DGKδ are involved in anchorage-independent growth and motility of Kaposi sarcoma cells in response to hepatocyte growth factor, respectively [182]. DGKζ, which is necessary for the growth and survival of glioma cells [183], promotes invasiveness [184] and lipogenic metabolism, as well as the growth of colon cancer cells [185].

### 5.2. Diabetes and Its Complications

DGK isoforms are reported to exhibit both pro-diabetic and anti-diabetic properties. The DAG-PKC pathway is involved in the pathogenesis of diabetic microvasculature complications [186]. The complications include nephropathy, neuropathy, and retinopathy that are associated with hyperglycemia (it may be necessary, but not sufficient to trigger the complications), oxidative stress, and mitochondrial dysfunction [187]. PKC activation decreases glucose uptake, upregulates the expression of profibrotic TGF-β1, and increases the production of the extracellular matrix, which contribute to the progression of renal insufficiency. As DGK negatively regulates PKC activation, dysfunction of several DGK isoforms may exacerbate diabetic phenotypes. The deficiency of DGKδ promoted hyperglycemia-induced peripheral insulin resistance and exacerbated the severity of type II diabetes [167]. Vitamin E (D-α-tocopherol)-activated DGKα is involved in the vitamin E-induced alleviation of diabetic nephropathy in vivo [73] and regulates the immune responses in the white adipose tissue [188]. DGKγ regulates insulin secretion [189].

Different DGK isoforms are reported to exhibit pro-diabetic properties. DGKε deficiency preserves glucose tolerance and increases lipid metabolism in obese mice [190]. DGKζ KO mice were protected against peripheral insulin resistance and showed increased respiratory exchange ratio [191]. These findings suggest the need to differentiate the non-redundant signaling pathways regulated by pro-diabetic and anti-diabetic DGK isoforms for developing potential diabetes therapeutics.

### 5.3. Cognition and Mood Impairment

Fragile X mental retardation protein (FMRP) regulates DGKκ expression in the neurons. In fragile X syndrome, a condition in which FMRP-dependent DGKκ translation does not occur, DAG generated from the activation of metabotropic glutamate receptor mGluRI, a GPCR, is not transformed into PA in neurons [168]. GWAS have suggested that DGKθ is associated with the pathogenesis of Parkinson’s disease [192,193].

Studies on KO mice have demonstrated that several DGK isoforms regulate brain function through the direct regulation of synaptic plasticity [194]. DGKβ is a learning-regulated gene in humans [195]. DGKβ KO mice exhibited impaired cognitive functions, including impaired spatial and long-term memories [196] and bipolar disorder-like phenotypes [196,197]. The administration of lithium alleviated the impairment of memory and emotion in the DGKβ KO mice [198], which suggested that the expression level of DGKβ is a putative biomarker for screening patients with lithium-responsive bipolar disorder.

DGKδ KO mice exhibit obsessive-compulsive disorder (OCD)-like phenotypes [199]. DGKη KO mice exhibited bipolar disorder (mania)-like phenotypes, which was alleviated upon lithium administration [200]. DGKε regulates seizure susceptibility and long-term memories [88]. Additionally, DGKε has been suggested to contribute to the pathogenesis of Huntington disease [201].

DGKζ is targeted to excitatory synapses through the direct interaction with postsynaptic density protein-95 (PSD-95). Previous studies have reported that DGKζ deficiency leads to decreased spine density [202], impaired bone resorption, and reduced muscle hypertrophy [157]. However, DGKζ gene mutations have not been reported in humans.

### 5.4. Inflammation and Immunity

The inhibition of DGKα and DGKζ enhances the T cell antitumor response and the efficacy of immunotherapies, such as chimeric antigen receptor-modified T (CAR-T) cell therapy [203]. Genetic ablation of DGKζ results in hyperresponsive NK cells, which exhibit increased cytokine production and degranulation through an ERK-dependent mechanism [110]. DGKα inhibition can restore the impaired functions of T cells and NK cells.

The targeted deletion of DGKζ in the T cells decreases type 2 inflammation without reducing airway hyperresponsiveness (AHR). However, the loss of DGKζ in the airway smooth muscle cells decreases AHR but not airway inflammation [204]. DGKζ was reported to limit the inflammatory cytokine production and pro-inflammatory M1-like macrophage polarization through the downregulation of STAT1 and STAT3 phosphorylation in the cytokine storm syndrome mouse model, which was dependent on TLR9 signaling, and the arthritic mouse model, which was dependent on TLR2 signaling [205]. The DGKζ-deficient mast cells exhibit impaired degranulation, increased IL-6 secretion, and upregulated Ras/AKT/ERK signaling [206]. DGKγ regulates the antigen-induced degranulation of the mast cells through the modulation of Ca^2+^ influx [207].

The activation of cPKC and RASGRP4 in the neutrophils, which are downstream effectors of DAG signaling, promotes NADPH oxidase activity, leukocyte movement, and extracellular trap release (NETosis) [104,208]. The activation of cPKC by PMA, a DAG analog, promotes NET formation. However, there are no in vitro or in vivo studies examining the direct regulation of NETosis by DGK. Additionally, there are limited studies on the biological role of DGKα in the immune cells other than the T cells. However, increasing evidence indicates that DGKα regulates leukocyte function [209]. The stimulation of human neutrophils with N-formylmethionine-leucyl-phenylalanine promotes DAG production, most of which is metabolized by DGKα. Treatment with R59022 enhances the DAG levels, which indicated that the inhibition of DGKα is required for neutrophil activation [210]. ANCAs target myeloperoxidase and proteinase 3 to promote adhesion, degranulation, and oxidative burst in the neutrophils. The neutrophils incubated with R59022 before treatment with ANCAs exhibited a decreased release of azurophilic granules containing cytotoxic factors, such as proteases and myeloperoxidase, which is caused due to the inhibition of granule fusion at the plasma membrane [163]. This inhibition is restored by PA treatment, which indicated that DGKα upregulates granule exocytosis.

## 6. Conclusions

Studies on the regulation of DAG/PA levels by various enzymes, the identification and characterization of various DAG/PA-regulated proteins, and the overlap of different DAG/PA metabolic and signaling processes have revealed the complex roles of DGKs in regulating multiple biochemical and biological networks. However, the existence of many isomeric forms of DAG/PA, non-redundant functions and versatile subcellular localization of DGKs, and multiple signaling pathways regulated by DGKs impede our efforts to delineate the specific contribution of each DGK isoform in maintaining cellular homeostasis. Thus, further studies are necessary to characterize the physiological functions of DGK isoforms in the cells of the tissue microenvironment to develop therapeutic agents for pathological conditions associated with dysregulated downstream oncogenic pathways, immunity, and angiogenesis.

Lipid metabolism is a dynamic, complex, and bi-directional process. Hence, the functions of DGK isoforms are also dependent on their interaction with other lipid-modifying enzymes. Currently, there are no known inhibitors of DGK isoforms, except DGKα. The known inhibitors of DGKα are not selective. In contrast, several inhibitors of PA-synthesizing LPAAT and PLD have been reported. The precise modulation of PA synthesis using a combination of inhibitors of multiple DGK isoforms and PA synthesis enzymes may improve the therapeutic efficacy against cancer. Further studies are needed to explore these therapeutic strategies.

However, the successful lab-to-clinic translation will need the identification of novel specific inhibitors of DGK isoforms and the elucidation of mechanisms that regulate the function and expression of DGK isoforms and signaling events comprising direct targets and effectors of DGK isoforms. The biological functions of DGK isoforms have been mostly explored in the immune and nervous systems, while lab-to-clinic translation efforts have mainly focused on cancer. Future studies must correlate laboratory and preclinical findings with the clinical findings.

We expect that the simultaneous functional comparisons of inhibition of DGK isoforms can delineate the specific contribution of each isoform in maintaining homeostasis, which will promote the restoration or maintenance of tissue microenvironment.

## Figures and Tables

**Table 1:** Expression pattern and subcellular localization of DGK isoforms.

Isoform	Main tissue Expression	Subcellular Localization
α	T cell [2], brain [4,80]	Plasma membrane and nucleus [24,76], cytoplasm [4,76]
β	Brain [5,81]	Cytoskeleton [6,24], perisynaptic membrane [81]
γ	Brain [6,7]	Golgi and nucleus [24,77]
δ	Ubiquitous [8,82,83,84]	Cytoplasm [8,24,60,85], endoplasmic reticulum [24], endosomes [86], nucleus [60]
η	Ubiquitous [9,83,84,87]	Cytoplasm [9,87], endosomes [24]
κ	Reproductive organs [10,83,84]	Plasma membrane [10,24],
ε	Ubiquitous [11,84,88]	Plasma membrane and endoplasmic reticulum [24,89]
ξ	Ubiquitous [12,84]	Cytoplasm [90], plasma membrane and nucleus [24,78,91]
ι	Brain [13,79], retina [13,92]	Cytoplasm [90], nucleus [24,79]
θ	Brain [14], smooth muscle [93] and endothelial cells [93]	Plasma membrane and nucleus [24,60], presynaptic vesicles [26]

## Abbreviations

ER	Endoplasmic reticulum
fMLP	N-formylmethionine-leucyl-phenylalanine
GPCR	G protein-coupled receptor
GSK3	Glycogen synthase kinase 3
HGF	Hepatocyte growth factor
HIF-1	Hypoxia-inducible factor 1
IκB	Nuclear factor of kappa light polypeptide gene enhancer in B cell inhibitor
IP3	Inositol 1,4,5-triphosphate
IQGAP	IQ motif-containing GTPase-activating protein
PIP2	Phosphatidylinositol (4,5)-bisphosphate
PI3K	Phosphatidylinositide-3-kinase
PLs	Phospholipids
PLC	Phospholipase C
mGluRI	Group I metabotropic glutamate receptors
MHC	Major histocompatibility complex
MTOC	Microtubule-organizing center
mTORC	Mammalian target of rapamycin complex
MVB	Multivesicular bodies
NFAT	Nuclear factor of activated T cells
NF-κB	Nuclear factor kappa-light-chain-enhancer of activated B cells
NO	Nitric oxide
ROS	Reactive oxygen species
SF1	Steroidogenic factor 1
SNX27	Sorting Nexin 27
SREBP1	Sterol regulatory element-binding protein 1
TF	Transcription factor
TLR	Toll-like receptor
TME	Tumor microenvironment
TPA	12-O-tetradecanoyl-phorbol-13-acetate
VEGF	Vascular endothelial growth factor
YAP/TAZ	Yes-associated protein and transcriptional coactivator with PDZ-binding motif
